# Loss of PIM1 correlates with progression and prognosis of salivary adenoid cystic carcinoma (SACC)

**DOI:** 10.1186/s12935-018-0518-y

**Published:** 2018-02-20

**Authors:** Jiajie Xu, Xin Zhu, Qingling Li, Chao Chen, Zhenying Guo, Zhuo Tan, Chuanming Zheng, Minghua Ge

**Affiliations:** 1 Department of Head and Neck Surgery, Zhejiang Cancer Hospital, No. 38 Guangji Road, Hangzhou, 310022 Zhejiang China; 2 Zhejiang Cancer Research Institute, Hangzhou, 310022 China; 3 Department of Medical Oncology, Zhejiang Cancer Hospital, Hangzhou, 310022 China; 4 Department of Pathology, Zhejiang Cancer Hospital, Hangzhou, 310022 China

**Keywords:** Salivary adenoid cystic carcinoma, PIM1, p21, RUNX3, Short hairpin RNA

## Abstract

**Background:**

Increasing evidence indicates that PIM1 is a potential prognostic marker and target for cancer treatment but its precise mechanisms of action remain to be determined in salivary adenoid cystic carcinoma (SACC). This study aims to decipher the prognostic and mechanistic role of PIM1 in progression of SACC cells and tumor tissues.

**Methods:**

A SACC cell line (ACC-M) was transfected with shRNA plasmids targeting the PIM1 gene. The expression levels of PIM1, RUNX3 and p21 were measured by quantitative real-time PCR and western blot. Subcellular translocalization of RUNX3 and p21 proteins was assessed using immunofluorescence, and cell cycle phase was quantified using flow cytometry. A total of 97 SACC patients were retrospectively analyzed by clinicopathologic characteristics and survival outcomes.

**Results:**

After down-regulation of PIM1 in ACC-M cells, RUNX3 and p21 proteins were translocated from cytoplasm to nucleus, with a decrease of p21 expression and increase of G0/G1 phase cells. PIM1 and RUNX3 levels show a distinct covariance. PIM1 is associated with T-status, lymph node involvement, nerve invasion, and distant metastasis in SACC tissues. Patients with low PIM1 level had a better outcome than those with higher PIM1 level.

**Conclusions:**

PIM1 is multifunctional in ACC-M cells and it serves as a neoteric therapeutic target and potential prognostic marker for SACC patients.

## Background

Adenoid cystic carcinoma (ACC), a common salivary gland cancer subtype, accounts for 22% of salivary gland malignancies in humans [1]. Salivary adenoid cystic carcinoma (SACC) is characterized by highly aggressive nerve and vessel infiltration, high rate of recurrence, and frequent metastasis to lung [2]. Local recurrence and distant metastasis resulting from tumor insensitivity to radiotherapy and chemotherapy are the primary causes for SACC therapy failure [3]. The main treatment includes local resection with additional radiation therapy and chemotherapy, a treatment regimen that has not changed over many years. Lack of novel and effective treatments to inhibit SACC progression is still a major hurdle to overcoming this deadly disease. It is important to further understand SACC progression and the molecular mechanisms driving SACC.

PIM1, identified as a preferential integration site for moloney murine leukemia virus to induce T-lymphomas in mice [4], belongs to a small group of kinases with two homologues (PIM2 and PIM3). PIM1 is an oncogene and its encoded product exhibits serine/threonine kinase activity [5]. In several human cancer types, including T-and B-cell leukemia and lymphomas, the overexpression of PIM1 inhibits apoptosis, promotes proliferation, and prevents differentiation and maturation [6]. PIM1 kinase promotes cell cycle progression and proliferation by phosphorylating and enhancing the phosphatase activity of G0 phase regulator Cdc25A and G2/M phase regulator Cdc25C [7].

PIM1 is thought to be constitutively active without the need for posttranslational modification; the level of its kinase activity therefore likely depends on the absolute amount of protein in cells [8]. The preferential phosphorylation consensus sequence of PIM1 is (Arg/Lys) 4-X-Ser/Thr-XV [9, 10]. Proteins phosphorylated by PIM1 include p21, p100, cdc25A phosphatase, HP1, PAP1, PTP-U2S, NFATc1, TFAF2/SNX6 and nuclear mitotic apparatus protein (NuMA) [11–19]. Previous studies demonstrated that overexpression of PIM1 could inhibit apoptosis of tumor cells by regulating the cell cycle [20, 21]. Inhibition of PIM1 expression suppresses growth of tumor cells [22]. Although PIM1 is a known oncogene in various human cancers, its potential role as a therapeutic target has not been explored in SACC. In the present study, the expression of PIM1 gene was inhibited in ACC-M cells by constructing short hairpin RNA (shRNA) against PIM1 to understand its role in SACC.

One potential mechanism for PIM1 in SACC cells is phosphorylation of p21. It is known that the p21 gene encodes a 21kD protein which binds and inhibits a wide range of cyclin/CDK complexes and predominantly blocks G1/S phase cell cycle transition. Also, p21 interacts with proliferating cell nuclear antigen (PCNA) in the nucleus to inhibit DNA replication [23, 24]. More exactly, the C-terminal portion of p21 is phosphorylated at Thr145 by PIM1 and this phosphorylation event results in p21 subcellular localization [11]. A study by Zhang et al. implies that PIM1 might influence cell cycle progression by affecting p21 [5].

The RUNX3 tumor suppressor gene encodes a transcription factor that regulates lineage-specific gene expression in developmental processes and is involved in the formation of multiple cancers. The expression level of RUNX3 can be a prognostic indicator in variety of malignancies [25]. Our previous study demonstrated that RUNX3 protein is mainly expressed in nucleus and cytoplasm of acinous cells and ductal cells in normal salivary gland, but only in cytoplasm of tumor cells in SACC. RUNX3 cytoplasmic expression levels may significantly affect prognosis [26]. The expression of RUNX3 protein could be a potential biomarker for diagnosis and prognosis of SACCs. In SACC cells, PIM1 may alter subcellular localization of RUNX3 by phosphorylation, which has been demonstrated in HEK 293 cells [27]. In addition, RUNX3 is the upstream regulator of p21 expression. It is clear that the tumor suppressor activity of RUNX3 is at least partly associated with its ability to induce p21 expression [28].

PIM1 is involved in multiple cellular activities and it is likely that PIM1 has more unidentified substrates. As we know, PIM1 regulates the subcellular localization of p21 [11] and RUNX3 [27], which may lead to cellular apoptosis in SACC. Increasing evidence indicates that PIM1 is a potential target for cancer treatment but its precise mechanisms of action remain to be determined in SACC. In the present study, we describe various modes of action that PIM1 exerts in SACC cells (ACC-M) in vitro. These results may assist in development of PIM1 inhibitors to treat SACC.

## Materials and methods

### Construction of pGPU6/GFP/Neo-shRNA carrier

The plasmid pGPU6/GFP/Neo was purchased from GenePharma and the negative control plasmid (no homology to disrupt the sequence) was provided by GenePharma. We designed and synthesized four pairs of inference shRNA oligos (Shanghai Invitrogen Biotechnology Co., Ltd, China). Annealed shRNA oligos were ligated into the pGPU6/GFP/neo vector before transformation into *E. coli*. We selected 3 clones from each plate for growth in LB liquid medium containing 50 mg/L kanamycin overnight at 37 °C. Clones were analyzed by sequencing (Shanghai Invitrogen Biotechnology Co., Ltd, China). The four target sites for PIM1 shRNA are shown below:

- PIM1-shRNA-1, 5′-AACATCCTTATCGACCTCAATCGCG-3′;
- PIM1-shRNA-2, 5′-GTCTCTTCAGAATGTCAGCAT-3′;
- PIM1-shRNA-3, 5′-GGATCCTGCTGTATGATATGG-3′;
- PIM1-shRNA-4, 5′-GGGTTTCTCCGGCGTCATTAG-3′.

### Transfection of ACC-M cells

ACC-M was purchased from the Cell Bank for Type Culture Collection, Chinese Academy of Sciences (Beijing, China), and cultured in RPMI-1640 medium (Gibco) supplemented with fetal calf serum (10% Hyclone), penicillin (100 U/mL) and streptomycin (100 μg/mL). ACC-M cells were seeded in a 6-well culture plate at a density of 1 × 10^5^ cells/well. After 24 h, transfection complexes were assembled as follows: 4 μg of pGPU6/GFP/Neo-shRNA plasmid or negative control plasmid was resolved in 400 μL RPMI 1640 medium (serum-free and antibiotic-free). To this, 9 μL Lipofectamine™ 2000 transfection reagent was added, thoroughly mixed, and allowed to stand at room temperature for 10 min to form transfection complexes. The transfection mix was added to wells and cells were cultured at 37 °C in a 5% CO_2_ incubator. The cells were collected 24–48 h after transfection.

### Reverse transcriptase (RT)-PCR and quantitative real-time PCR (qRT-PCR)

RT-PCR and qRT-PCR were performed as described previously [29]. The following primers were used:

- PIM1 forward, 5′-TGTGCTGGGAGAAATACTTGAA-3′ and reverse, 5′-AGGTGGCTCAGCGTTTGG-3′ (size: 160 bp);
- p21 forward, 5′-GGAGACTCTCAGGGTCGAAAACG-3′ and reverse, 5′-GAGAAGATCAGCCGGCGTTTG-3′ (size: 77 bp);
- RUNX3 forward, 5′-CCAAGGCACCTCGGAACTGAAC-3′ and reverse, 5′-TCTCCGTGAGGGTTGGCAGC-3′ (size: 83 bp);
- GAPDH forward, 5′-CATGAGAAGTATGACAACAGCCT-3′ and reverse, 5′-AGTCCTTCCACGATACCAAAGT-3′ (size: 113 bp).

After PCR amplification, dissociation of SYBR Green-labeled cDNA (melt curve analysis) was carried out to affirm that there were no nonspecific PCR products. 2^−∆∆Ct^ method was performed to analyze the relative quantification of transcript expression.

### Detection of protein expression by western blot

Cells were harvested at 80% confluence and lysed by suspension in RIPA lysis buffer followed by gentle sonication. Protein lysates (20 μg) were separated on 8% SDS polyacrylamide gel by electrophoresis and transferred to PVDF membrane (Millipore). Membranes were then blocked in PBS/Tween (PBS with 5% non-fat powered milk and 0.05% Tween 20) at room temperature for 2 h. The blot was then incubated with affinity-purified rabbit anti-PIM1 (Novus NBP1-40501, 1:500) overnight. The membranes were washed 3–4 times with PBS/Tween and incubated with HRP-conjugated goat anti-rabbit IgG (JIR 111-035-003, 1:8000) for 2 h at room temperature. After repeating the washing steps, the signal was detected by chemiluminescence using the Pierce Super Signal West Pico Systems (Pierce).

### Cell cycle analysis

ACC-M cells (1 × 10^6^/mL) in logarithmic growth phase were plated in 6-well plates and transfected as described above. The cells were fixed with ice-cold 70% ethanol at − 20 °C for 12–24 h. The cells were collected after centrifugation and resuspended in 500 μL of PBS buffer containing RNase A (the final concentration of RNase A was 0.25 mg/mL) and incubated at 37 °C for 30 min. Five microliter PI was added and incubated for 30 min at room temperature in dark. Data were acquired by flow cytometry (FACS Calibur, Becton–Dickinson).

### Immunofluorescence staining analysis

ACC-M cells were transfected as described. After 48 h, cells were washed twice with PBS. Following fixation with 4% paraformaldehyde (15 min, 37 °C), cells were washed three times in PBS and then permeabilized with 0.1% Triton X-100 in PBS for 2 min at room temperature. Samples were blocked with 3% BSA and 0.05% Tween 20 in PBS (blocking solution) for 30 min at room temperature and then incubated overnight at 4 °C with primary antibodies (PIM1, Novus NBP1-40501, 1:200; p21, Novus NBP200-303, 1:200; RUNX3, Novus NBP1-46694, 1:200), respectively. For the immunofluorescence staining method, cells were incubated with secondary antibodies (1 µg/mL), DyLight 488 AffiniPure Goat Anti-Mouse IgG (H+L) (EarthOx) at 1:100 for Novus NB200-303 and DyLight 594 AffiniPure Goat Anti-Rabbit IgG (H+L) (EarthOx) at 1:100 for Novus NBP1-40501 and Novus NBP1-46694, There secondary antibodies were conjugated to Alexa-488 green fluorescence (Molecular Probes, Eugene, OR). Cells were counterstained with 4′,6-diamidino-2-phenylindole (DAPI, Sigma). Samples were visualized by confocal microscopy at 63× magnification.

### Tissue specimens

Ninety-seven patients with histopathologically proven SACC in Zhejiang Cancer Hospital between July 2002 and July 2014 were recruited for this study. The study was approved by the Ethics Committee of Zhejiang Cancer Hospital. All patients have signed the informed consent.

### Immunohistochemistry

Paraffin-embedded tissues were cut for 4 μm sections mounted on glass slides (Mats-unami,MS-coated glass) and dried overnight at 37 °C. After deparaffinization, antigen retrieval in 0.01 M citrate buffer, and inactivation of endogenous peroxidase activity in 3% H_2_O_2_/methanol, the slides were incubated with antibody for PIM1 (Novus NBP1-40501, 1:200) and RUNX3 (Novus NBP1-46694, 1:200) at 4 °C overnight. The immunoreactivity was visualized by using a streptavidin–biotin–peroxidase staining kit (Nichirei, Histofine Simple Stain Max PO Multi) and DAB solution (Nichirei, Simple Stain DAB). The results were presented as percentage of nucleus staining positive cells relative to total cells. The scores of staining results were given as negative and positive. In brief, IHC score was determined by combining staining frequency and intensity. In detail, the staining frequency score was defined as no cell stained scored as 0, 1–10% of cells stained as 1, 11–50% of cells stained as 2, 51–80% of cell stained as 3, and 81–100% of cell stained as 4. Staining intensity score was rated on a scale of 0–3, with 0 for negative; 1 for weak; 2 for moderate; and 3 for strong staining. Theoretically, the scores could range from 0 to 12. An IHC score of 9–12 was considered as strong immunoreactivity, 5–8 as moderate, 1–4 as weak and 0 as completely negative. Sections in which the staining could not be well characterized were considered as equivocal. Staining was scored independently by two pathologists who were blinded to each other’s findings. All conflicting calls on scoring were adjudicated by a third individual. We used IHC score 5–12 for normal to high expression (positive) and 0–4 for no to low expression (negative).

### Statistical analysis

SPSS 22.0 was used to perform one-way ANOVA for the discrepancy of three groups or more. The two-tailed Student’s t-test was used to detect any statistically significant difference between two groups. Associations between PIM1, RUNX3 levels, and clinicopathologic parameters were analyzed by using the χ^2^ test or the Fisher exact test. Survival analysis was carried out by the Kaplan–Meier method and significant differences were assessed by means of the log-rank test. p values < 0.05 were considered to be statistically significant.

## Results

### Downregulation of PIM1 in ACC-M cells treated with pGPU6/GFP/Neo-shRNA

In this study, we used Lipofectamine™ 2000 reagent to optimize the transfection condition. The pGPU6/GFP/Neo-shRNA vector can express green fluorescent protein after being transfected into ACC-M cells. By fluorescence microscopy, we found that the total transfection rate was more than 70% after transfected for 48 h.

**Fig. 1:**
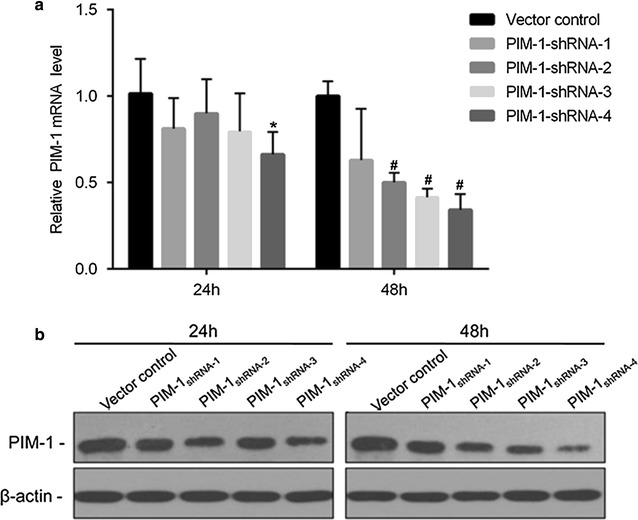
PIM1-shRNA induced PIM1 gene silencing in ACC-M cells. ACC-M cells were transfected with 4 μg of shRNA expression plasmids against PIM1 (RNAi) or with shRNA-NC (vector control). RNAi and control samples were collected at 24 and 48 h and analyzed with real time-PCR (**a**) and western blotting for the expression of PIM1 (**b**). *GAPDH* was amplified as the real time PCR internal control, β-actin was used as the loading control for western blotting. **a** *Significantly different compared to control in 24 h (*p* < 0.05) and ^#^significantly different compared to control group in 48 h (*p* < 0.05). Vector control: negative control for transfection of non-targeting sequence; PIM1-shRNA-1, PIM1-shRNA-2, PIM1-shRNA-3 and PIM1-shRNA-4 are small hairpin RNA targeted against PIM1

Both PIM1 mRNA and protein levels were significantly decreased in ACC-M cells after shRNA transfection. As shown in Fig. 1, real-time PCR results indicated that transcript levels of PIM1 in ACC-M cells were depleted by shRNA transfection (*p* < 0.05) in all interference groups. The PIM1-shRNA-3 transfection achieved the most effective knockdown of PIM1. Western blot analysis showed that the protein expression of PIM1 in ACC-M transfected with shRNA for 24 and 48 h was also decreased. PIM1-shRNA-3 and PIM1-shRNA-4 were most effective and thus used in the following experiments.

### Effects of pGPU6/GFP/Neo-shRNA transfection on the expression of p21 and RUNX3

**Fig. 2:**
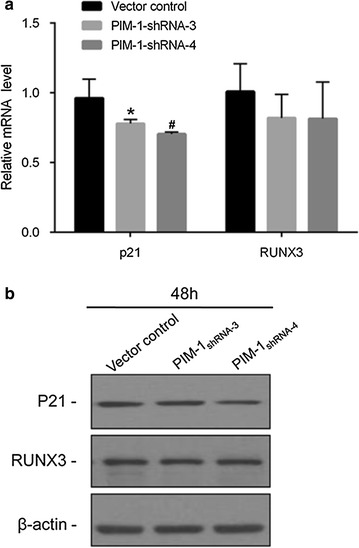
Knockdown of PIM1 depleted p21 transcript and protein. The mRNA and protein from shRNA-NC-transfected, and shRNA-PIM1-transfected cells were analyzed by real-time PCR (**a**) and western blot (**b**) to detect the expression of p21 and RUNX3. *Significantly different compared to control in 48 h (*p* < 0.01) and ^#^significantly different compared to control in 48 h (*p* < 0.01)

As shown in Fig. 2a, transcript levels of p21 were significantly decreased in ACC-M cells after transfection of shRNA targeting PIM1 (p < 0.05). There was no change in RUNX3 mRNA levels after PIM1 knockdown. Protein expression of p21 in shRNA transfected ACC-M cells was also decreased. There was no significant reduction of RUNX3 protein in the shRNA interference transfections versus the vector control group.

### Cell cycle analysis of ACC-M cells after transient RNAi silencing of PIM1

**Fig. 3:**
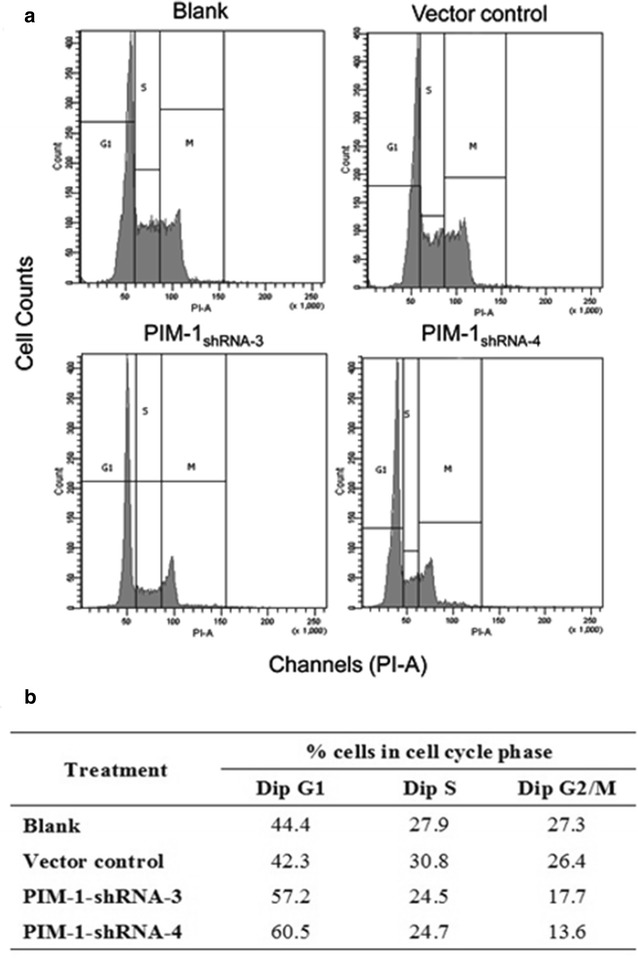
Downregulation of PIM1 induces ACC-M cell cycle arrest in G0/G1 phase. ACC-M cells transfected with the indicated shRNA-PIM1 or with scrambled control duplex (shRNA-NC) were cultured for 48 h and the use of DNA dye PI generates characteristic cellular DNA content profiles. **a** The PIM1-shRNA transfected ACC-M cells caused inhibition of the G1 stage, histograms represent the percentage of ACC-M cells in G1, S, and G2 phases of the cell cycle 48 h after shRNA-transfection. **b** Table representing percentages of cells in G0/G1, S and G2/M phases of the cell cycle after indicated transfection (treatment). Blank: non-transfected normal cell sample; vector control: negative control group; PIM1-shRNA-3 and PIM1-shRNA-4 represent PIM1 knockdown

Transfection of ACC-M cells with shRNA against PIM1 significantly disrupted the cell cycle. In the PIM1-shRNA-3 transfections, cells in G0/G1 stage increased from 42.3 to 57.2% (*p* < 0.05), while cells in G2/M stage and S stage decreased from 26.4 to 17.7% and 30.8 to 24.5%, respectively. Transfection with PIM1-shRNA-4 produced similar results; cells in G0/G1 stage increased from 42.3 to 60.5% (*p* < 0.05), while cells in G2/M stage and S stage decreased from 26.4 to 13.6% and 30.8 to 24.7%, respectively. These data indicated that shRNA transfection in ACC-M cells caused inhibition in the G1 phase, thus resulting in decreased numbers of cells in the S phase (Fig. 3).

### PIM1 knockdown causing translocalization of p21 and RUNX3 from cytoplasm to nuclear

**Fig. 4:**
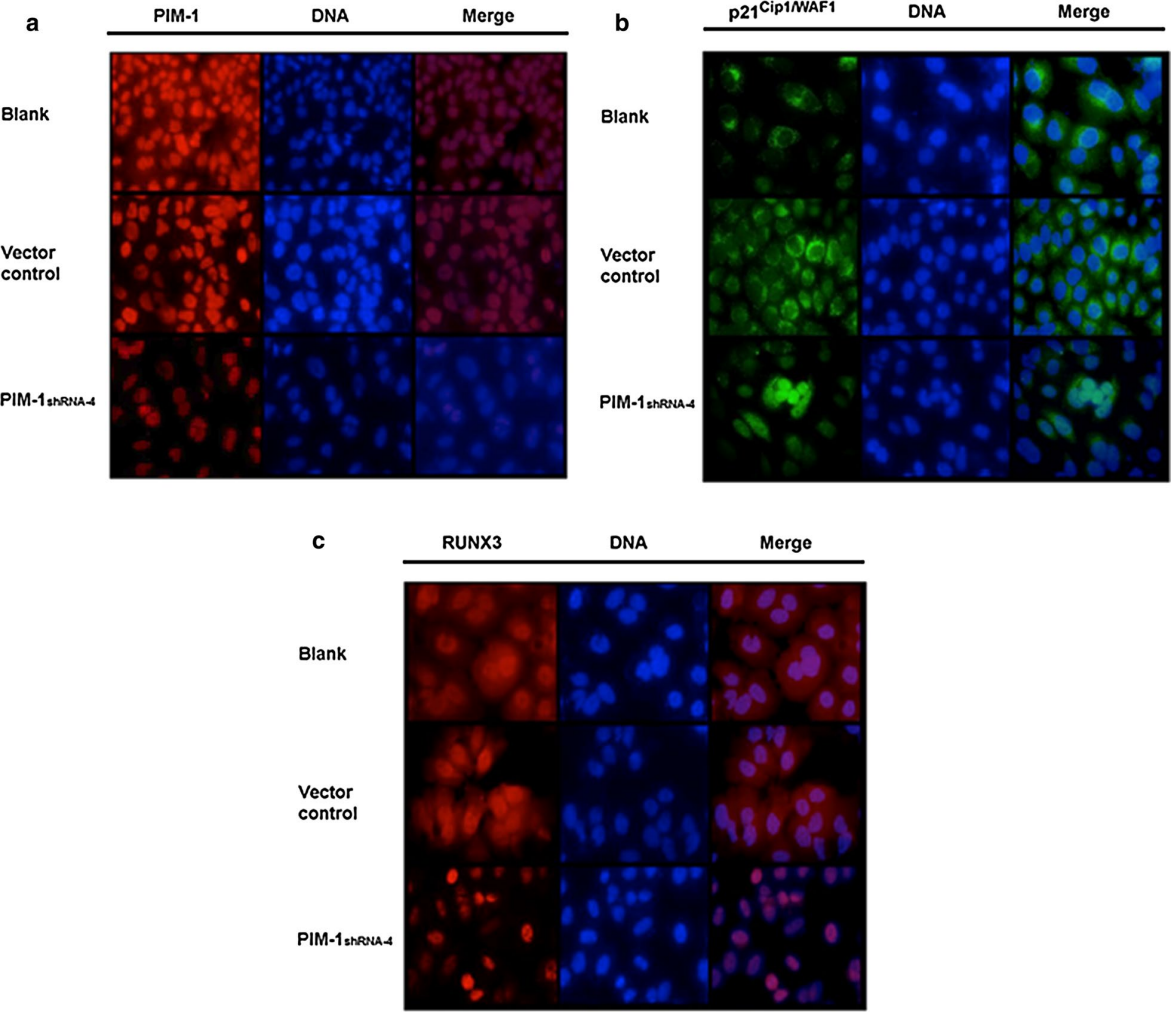
PIM1 altered the cellular localization of p21 and RUNX3. The localization of p21 and RUNX3 was visualized by immunostaining with anti-p21 and anti-RUNX3 antibodies. When shRNA-NC was transfected into ACC-M cells, p21 and RUNX3 were localized in the cytoplasm. However, when PIM1-shRNA-4 was transfected, p21 and RUNX3 were localized in the nucleus. **a** The PIM1 protein is expressed in ACC-M cells. **b** The p21 protein is predominantly cytoplasmic in control ACC-M cells, while p21 is almost exclusively localized to the nucleus 48 h after PIM1 knockdown. **c** Similarly, RUNX3 protein is nuclear and diffuse cytoplasmic in control ACC-M cells, but almost restricted to the nucleus upon PIM1 knockdown. Blank: non-transfected normal cell sample; vector control: negative control transfection of non-targeting shRNA; PIM1-shRNA-4 is for PIM1 knockdown. All images are captured at ×400

As shown in Fig. 4, p21 and RUNX3 are mainly located in the cytoplasm in the presence of PIM1. After transfection with shRNA against PIM1, p21 and RUNX3 staining is predominantly nuclear.

### Correlation between PIM1 and RUNX3 protein levels in SACC tissues

**Fig. 5:**
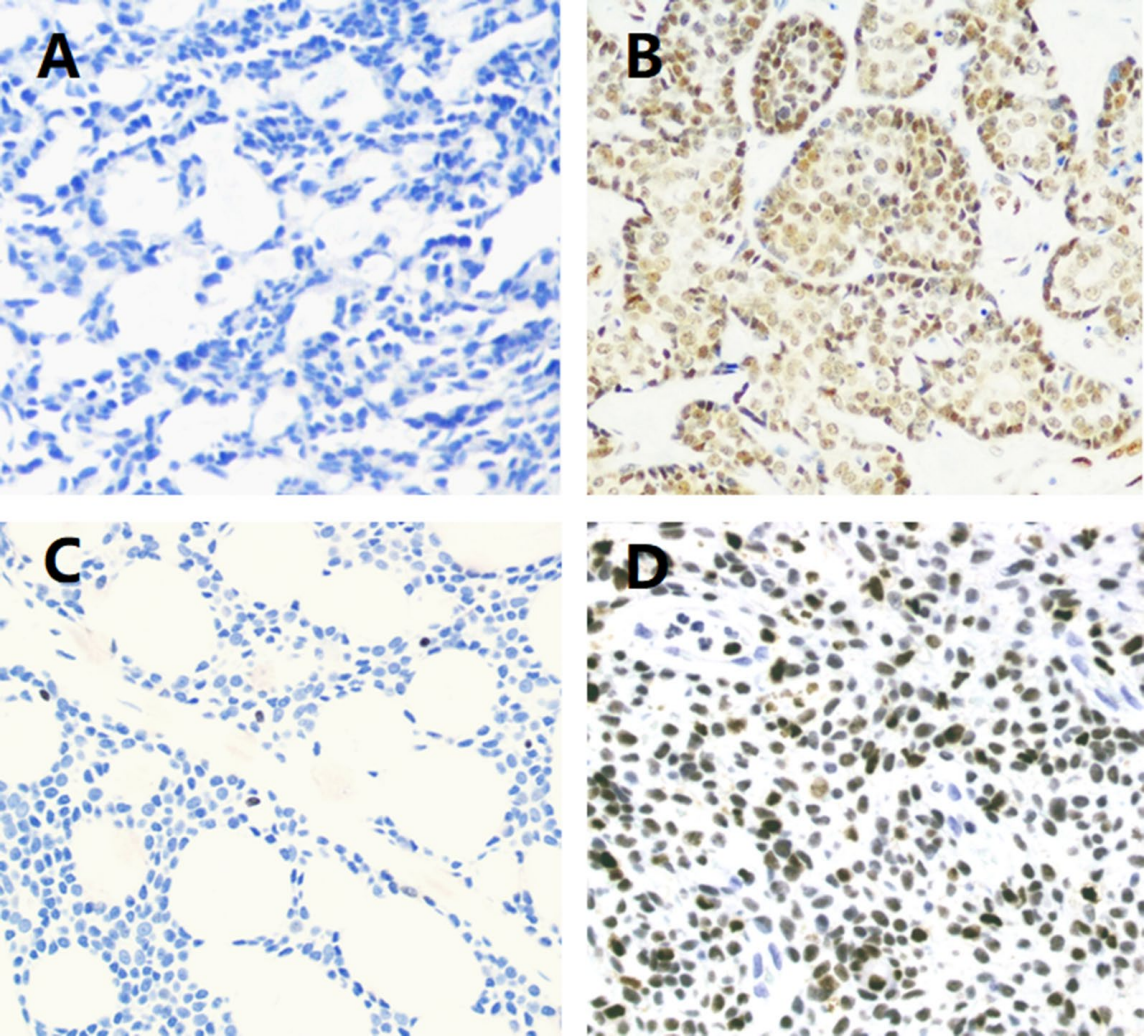
Immunohistochemical (IHC) staining of PIM1 and RUNX3 in SACC tissues (×400). **A** Negative PIM1 IHC staining in SACC tissues. **B** Positive PIM1 IHC staining in SACC tissues. **C** Negative RUNX3 IHC staining in SACC tissues. **D** Positive RUNX3 IHC staining in SACC tissues

PIM1 and RUNX3 immunohistochemical (IHC) staining in SACC tissues is shown in Fig. 5. PIM1 and RUNX3 positive ratios were 84.54% (82/97) and 18.56% (18/97), respectively. Table 1 showed a significant inverse correlation between the PIM1 and RUNX3 protein expression.

**Table 1:** Relationship between PIM1 and RUNX3 levels

Variable	Patients (n = 97)		*p*-value
	Negative	Positive	
PIM1	15	82	0.000
RUNX3	79	18	

### Correlation between the level of PIM1, RUNX3, and clinical characteristics in SACC tissues

We analyzed the correlation between PIM1 and RUNX3 expression in SACC tissues and clinicopathologic indexes. Table 2 shows that both PIM1 and RUNX3 levels were closely associated with T-status, lymph node involvement, nerve invasion, and distant metastasis (*p* < 0.05).

**Table 2:** Relationship between PIM1 and RUNX3 levels with the clinical characteristics

Variable	Patients (total = 97)		PIM1		*p*-value	RUNX3		*p*-value
			Negative	Positive		Negative	Positive	
Gender	Male	39	5	34	0.555	33	6	0.510
	Female	58	10	48		46	12	
Age	< 52	52	9	43	0.589	41	11	0.479
	≥ 52	45	6	39		38	7	
T-status	T1–2	33	14	19	0.000	20	13	0.000
	T3–4	64	1	63		59	5	
Tumor location	Major salivary	36	6	30	0.801	29	7	0.863
	Minor salivary	61	9	52		50	11	
Histological type	Cribriform	40	6	34	0.594	35	5	0.153
	Tubular	17	4	13		11	6	
	Solid	40	5	35		33	7	
Lymph node involvement	Yes	27	0	27	0.009	26	1	0.019
	No	70	15	55		53	17	
Perineural invasion	Yes	57	1	56	0.000	52	5	0.003
	No	40	14	26		27	13	
Distant metastasis	Yes	21	0	21	0.036	20	1	0.109
	No	76	15	61		59	17	

### Survival analysis

**Fig. 6:**
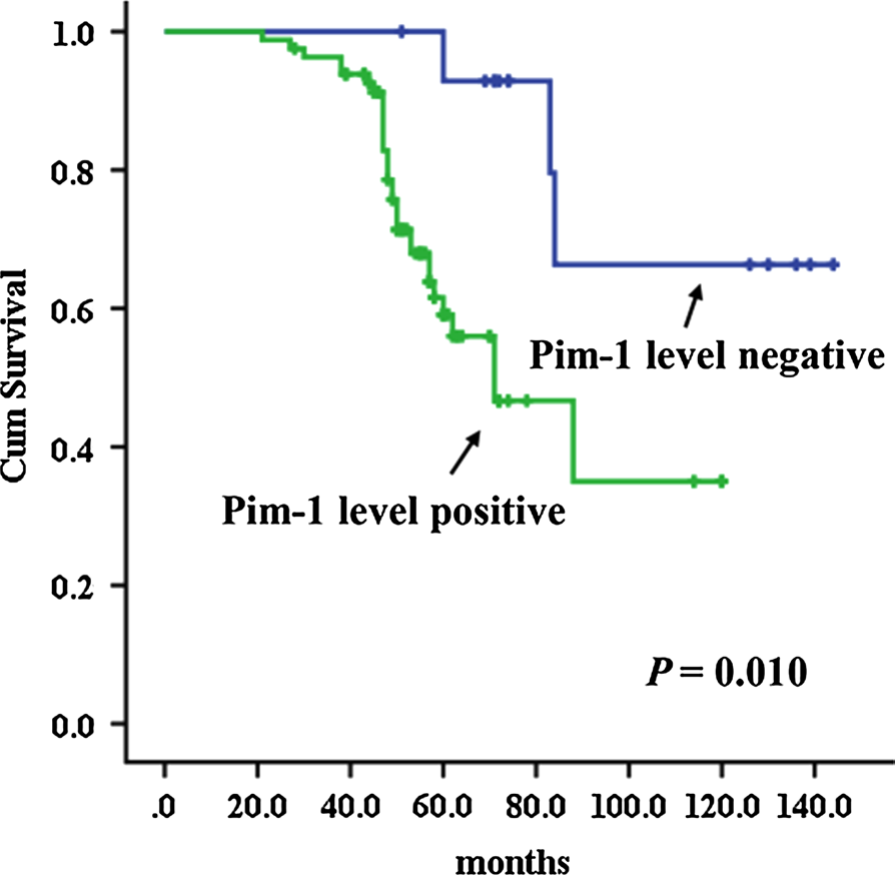
Two Kaplan–Meier curves for the relationship between PIM1 protein level and survival time of SACC patients

Kaplan–Meier survival curves (Fig. 6) indicate that high PIM1 levels had an intimate association with survival of SACC patients (*p* = 0.01). Patients with low PIM1 levels had much better outcome than those with higher PIM1 levels.

## Discussion

The PIM1 oncogene has been implicated in various human cancers including lymphomas, gastric, colorectal, and prostate carcinomas [30]. Highly conserved serine/threonine kinase activity encoded by PIM1 has been demonstrated to have an important role in regulating cell proliferation, differentiation, apoptosis, and tumorigenicity.

In this study, the pGPU6/GFP/Neo-shRNA was used as an optimized expression vector together with green fluorescent protein (GFP) reporter gene to deplete PIM1 levels in SACC cells. Using RNA interference technology, we successfully constructed shRNA recombinant plasmids targeting PIM1 gene expression. After transfection, silencing of PIM1 gene in ACC-M cells was evidenced by decreased PIM1 mRNA and protein expression. Silencing of the PIM1 gene caused ACC-M cell cycle arrest in G1 phase and decreased the proportion of cells in S phase.

Similar to other tumor types, interactions exist between oncogenes and tumor suppressor genes involved in the development and maintenance of SACC. Here, we propose a model wherein PIM1 kinase affects expression and subcellular localization of tumor suppressor genes in SACC. Our model is supported by data showing two suppressor genes, p21 and RUNX3, are affected by PIM1 kinase knockdown in ACC-M cells. The p21 protein is one of the most studied inhibitors of cell cycle-dependent kinase (CDK). p21 arrests the cell cycle in G1 phase by inhibiting the CDK activity in G0 phase [31]. RUNX3 gene is a well-studied tumor suppressor gene, and it is a downstream transcription factor of transcription growth factor-β (TGF-β) signaling pathway. TGF-β can effectively inhibit cell growth and disruptions of the TGF-β signaling pathway can lead to a variety of tumors [32]. RUNX3, a MST (mammalian sterile 20-like) signaling pathway terminal effector, assisted by MST and SAV1, can induce cell apoptosis [33]. Early in 2006, a research team identified novel protein–protein interactions between the C-terminal of human RUNX3 and PIM1 using yeast two-hybrid technology [34]. Previous study has demonstrated that PIM1 can phosphorylate four Ser/Thr residues within the Runt domain and stabilize RUNX3 protein [27]. In SACC tissues, we found that there was a significant inverse correlation between the PIM1 and RUNX3 expression.

The present study indicated that the mRNA and protein expression of PIM1 and p21 was decreased in pGPU6/GFP/Neo-shRNA-PIM1 transfected cells, while the mRNA and protein expression of RUNX3 was not affected. The knockdown of PIM1 induced nuclear localization of p21 and RUNX3. The p21 and RUNX3 proteins are involved in multiple cellular functions, especially in cell differentiation and apoptosis. Most of these functions depend on the interaction of p21 and RUNX3 with associated proteins in distinct cellular compartments. The redistribution of p21 and RUNX3 in ACC-M cells may activate cellular apoptosis. Our data suggests that silencing PIM1 regulates p21 and RUNX3 subcellular distribution and influences multiple cellular functions.

The IHC results show that a higher PIM1 and lower RUNX3 level is associated with advanced T-stage, lymph node involvement and nerve invasion that translates to aggressive tumor behavior. Importantly, other clinical features including gender, age, tumor location and histological grade type showed no correlation. Higher PIM1 levels are significantly associated with distant metastasis. This evidence suggests the important role of PIM1 in SACC, most likely mediated by RUNX3.

The short hairpin RNA expression vector for PIM1 silencing was successfully constructed, and pGPU6/GFP/Neo-shRNA-PIM1 could knockdown the expression of PIM1 gene and regulate the cell cycle. The tumor suppressors p21 and RUNX3 were affected by PIM1 kinase knockdown, which may provide a partial explanation for the pleiotropic effects of this multifunctional kinase. Our study indicates that PIM1 could be a potential prognostic marker for SACC and provides an experimental basis for SACC target therapy although the mechanism needs further elaboration and exploration. Therefore, more studies are needed in order to define mechanisms of PIM1 expression and function in SACC.

## Conclusions

Our studies demonstrate that down regulation of PIM1 expression effectively decreases p21 expression and disrupts cell cycle. The subcellular re-localization of RUNX3 and p21 caused by PIM1 knockdown may partially explain diverse cellular functions of PIM1. PIM1 serves as a neoteric therapeutic target and potential prognostic marker for SACC patients.

## Abbreviations

SACC: salivary adenoid cystic carcinoma
IHC: immunohistochemistry
GFP: green fluorescent protein
p21: p21CIP1/WAF1
RUNX3: runt-related transcription factor 3
RT-PCR: real time quantitative polymerase chain reaction
